# TRPV1 in Cardiovascular Disease: A Molecular Nexus of Treatment

**DOI:** 10.3390/biom16030344

**Published:** 2026-02-25

**Authors:** Qi Lu, Xiaoqing Ding, Binghong Gao

**Affiliations:** 1 School of Exercise and Health, Shanghai University of Sport, Shanghai 200438, China; 2421517019@sus.edu.cn; 2 School of Athletic Performance, Shanghai University of Sport, Shanghai 200438, China; 2311811002@sus.edu.cn; 3 Faculty of Health Sciences and Sports, Macao Polytechnic University, Macao 999078, China

**Keywords:** TRPV1, cardiovascular disease, myocardial infarction, ischemia–reperfusion injury, cardiac remodeling, heart failure, hypertension, diabetes

## Abstract

Cardiovascular disease (CVD) remains the leading cause of morbidity and mortality worldwide, necessitating a deeper understanding of novel regulatory mechanisms and therapeutic targets. Transient receptor potential vanilloid subtype 1 (TRPV1), a non-selective cation channel extensively expressed in the cardiovascular system, has been implicated in the pathogenesis and progression of various CVDs, including myocardial infarction, ischemia–reperfusion injury, adverse cardiac remodeling, heart failure, hypertension, and diabetes. Recent studies demonstrate that TRPV1 modulates key signaling pathways associated with inflammation, oxidative stress, mitochondrial function, and apoptosis, exerting both protective and detrimental effects depending on specific disease contexts and experimental conditions. The dual regulatory roles of TRPV1, mediated through pathways such as TRPV1/CGRP/SP and TRPV1/eNOS/NO, underline its complexity and clinical relevance. This review summarizes current findings on the expression and function of TRPV1 in diverse cardiovascular tissues and models, critically evaluates its role in CVD pathophysiology, and discusses the therapeutic potential of modulating TRPV1-associated signaling. Understanding these mechanisms may provide valuable insights into developing precise intervention strategies against cardiovascular diseases.

## 1. Introduction

Cardiovascular disease (CVD) is a leading cause of death and disability worldwide. In 2021, approximately 20.5 million people died from CVD, representing about one-third of all global deaths. CVD encompasses multiple conditions, including ischemic heart disease, pathological cardiac remodeling, heart failure, as well as associated hypertension and diabetes. According to data from 2023, ischemic heart disease remains the foremost cause of death globally, particularly in low- and middle-income countries [1]. The development of ischemic heart disease is frequently linked to vascular dysfunction, mitochondrial dysfunction, and apoptosis, which are often induced by the rapid rise in intracellular calcium ion concentration during reperfusion. Both high dietary sodium intake and prolonged excessive workload on the heart are correlated with myocardial hypertrophy and fibrosis. Initially, this remodeling process is functional, compensatory, and adaptive. However, with persistent adverse stimuli, it progresses to pathological structural changes. These alterations can become self-sustaining and may ultimately trigger the onset of arrhythmias, hypertension, and other diseases [2,3].

Transient receptor potential vanilloid (TRPV) channels are extensively involved in numerous physiological and pathological processes, including pain perception, thermoregulation, inflammatory responses, and cardiovascular function. Among these, transient receptor potential vanilloid 1 (TRPV1), a member of the TRPV family, has emerged as a prominent research target in the cardiovascular field in recent years, owing to its role as a non-selective cation channel with significant calcium ion permeability [4]. Within the cardiovascular system, TRPV1 channels are primarily expressed in the ventricular wall, epicardial surface, sensory neurons innervating the myocardium, endothelial cells, and vascular smooth muscle cells. These channels can be activated by various physical and chemical factors [5]. TRPV1 modulates the progression and outcomes of cardiovascular diseases. Numerous studies have demonstrated a close association between TRPV1 and ischemic cardiac stimulation, as well as pathological cardiac remodeling induced by various factors. Nevertheless, current research findings remain inconsistent [2]. Evidence suggests that activation of the neuronal TRPV1 receptor exerts endogenous protective effects in myocardial ischemia–reperfusion injury by promoting the release of calcitonin gene-related peptide (CGRP) and substance P (SP) [6]. Conversely, other studies indicate that activation of TRPV1 in the dorsal root ganglion exacerbates myocardial injury following ischemia–reperfusion. These conflicting observations are also seen in studies investigating TRPV1 and pathological myocardial hypertrophy and fibrosis [7]. Additionally, research has shown that capsaicin can activate the peroxisome proliferator-activated receptor δ (PPAR-δ) signaling pathway via TRPV1, thereby ameliorating high-salt diet-induced myocardial hypertrophy and fibrosis [8]. However, studies have also demonstrated that mice lacking functional TRPV1 exhibit improved cardiac function under conditions of stress overload, along with reduced cardiac hypertrophy, fibrosis, and markers of apoptosis [9].

Overall, although the association between TRPV1 and cardiovascular diseases is widely recognized, its specific role in the related pathogenesis and underlying molecular mechanisms remains unclear. The existing literature on the involvement of TRPV1 in the pathogenesis of these diseases and their molecular mechanisms is limited and has yielded inconsistent findings. Therefore, this review summarizes and analyzes the current state of research regarding the role of TRPV1 in ischemic heart disease—particularly myocardial infarction and ischemia–reperfusion injury—pathological cardiac remodeling, heart failure, as well as hypertension and diabetes, thereby providing a theoretical foundation for the treatment of cardiovascular diseases.

## 2. Structure and Function of TRPV1

TRPV1 is a non-selective cation channel composed of six transmembrane domains, with its N-terminal and C-terminal regions playing crucial roles in sensing and transmitting signals [10]. As a multimodal nociceptor, TRPV1 can be activated by physical stimuli such as low molar osmotic pressure, high temperatures (>43 °C), low pH (<6.0), as well as various endogenous and exogenous agents [5,11]. TRPV1 is highly expressed in the brainstem, midbrain, hypothalamus, and limbic system of the central nervous system, and is also widely distributed in peripheral tissues, including the heart, adipose tissue, and skeletal muscle [12]. At the cellular level within the cardiovascular system, TRPV1 is typically localized on the cell membrane in its tetrameric form [13]. It is expressed in the ventricular wall, on the epicardial surface, in sensory neurons innervating the myocardium, as well as in endothelial cells and vascular smooth muscle cells [4]. It should be noted that the expression of TRPV1 in cardiomyocytes remains controversial. Some studies have failed to detect TRPV1 expression in cardiomyocytes in the ventricles and atria of TRPV1cre-tdTomato mice. Conversely, other research has demonstrated colocalization of TRPV1 with the mitochondrial-specific protein TOM20 (translocase of the outer mitochondrial membrane 20) in primary neonatal rat cardiomyocytes, leading to the hypothesis that TRPV1 might be present within cardiomyocyte mitochondria [14]. This view is supported by biochemical evidence, as another study detected the presence of TRPV1 protein via Western blot analysis in cardiac mitochondrial fractions isolated by differential centrifugation [15]. However, the hypothesis regarding the mitochondrial localization of TRPV1 remains to be definitively confirmed. Key challenges include the lack of direct electrophysiological evidence from mitochondrial membranes and the absence of TRPV1 in major mitochondrial protein databases such as MitoCarta. This discrepancy may be attributed to differences in antibody specificity and sensitivity used in the assays, as well as insufficient signal-to-background ratios in conventional immunofluorescence methods [16]. As a non-selective cation channel, TRPV1 permits the passage of cations such as H^+^, Na^+^, Ca^2+^, and Mg^2+^, exhibiting higher permeability to Ca^2+^ [5]. In recent years, TRPV1 has emerged as a novel target in cardiovascular research, primarily by initiating a series of critical physiological functions through the promotion of Ca^2+^ influx.

Common endogenous agonists of TRPV1 include anandamide, N-arachidonoyldopamine, leukotriene B4, oleoylethanolamide, palmitoylethanolamide, and N-oleoyldopamine. Exogenous agonists comprise capsaicin, resiniferatoxin (RTX), cannabidiol, piperine, evodiamine, and gingerol [17]. These agonists modulate cellular physiological functions by binding to specific sites on TRPV1, inducing conformational changes that open the channel’s ion pore. For example, vanilloids such as capsaicin and RTX act on the vanilloid-binding pocket of the TRPV1 channel, stabilizing the outward-facing conformation of the S4-S5 linker via interactions with specific amino acid residues. This conformational shift drives the movement of the S6 segment, transitioning TRPV1 from a closed to an open state and allowing cations such as Ca^2+^ to enter the cell through the channel [18].

Upon activation of TRPV1, a substantial influx of Ca^2+^ depolarizes nerve endings and triggers the generation of action potentials [19]. This process promotes intracellular signaling cascades and neurotransmitter release, thereby regulating cardiovascular function. The regulatory effects of TRPV1 are closely related to its cellular localization. For example, in sensory nerve fibers innervating mesenteric resistance arteries, TRPV1 activation induces the release of the vasoactive neuropeptide substance P (SP), which binds to neurokinin-1 (NK1) receptors to elicit smooth muscle contraction and vasoconstriction, thereby contributing to hypertension management [20]. In contrast, in sensory neurons and vascular endothelium, TRPV1 activation leads to vasodilation by promoting the release of calcitonin gene-related peptide (CGRP) [21] or by activating nitric oxide synthase (NOS) to produce nitric oxide (NO) [22]. Thus, CGRP and SP are key downstream neuropeptides activated by TRPV1, and their release is crucial for maintaining normal cardiovascular function.

**Figure 1:**
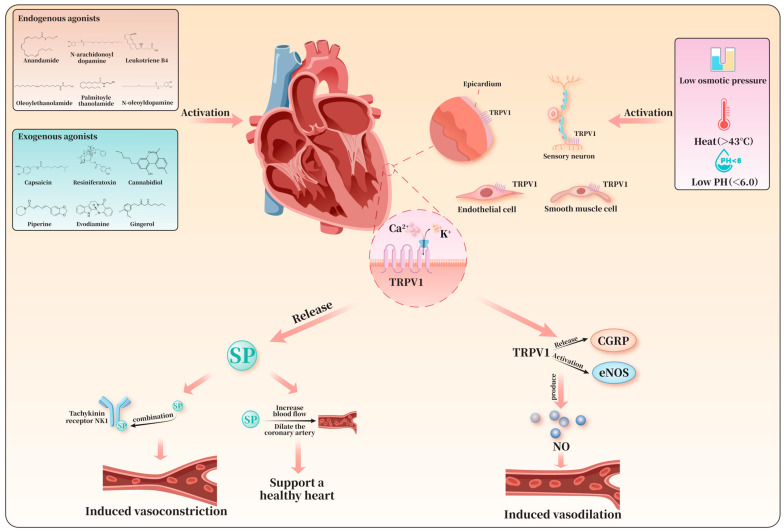
Mechanism of TRPV1 activation in the cardiovascular system. TRPV1: transient receptor potential vanilloid 1; SP: substance P; CGRP: calcitonin gene-related peptide; eNOS: endothelial nitric oxide synthases.

Previous studies have demonstrated that intracoronary infusion of SP can exert cardioprotective effects by increasing blood flow and dilating epicardial coronary arteries [23]. CGRP, in turn, mitigates endothelin-1-induced myocardial injury through the activation of myocardial protein kinase C (PKC) [24]. In addition, TRPV1 also serves as a regulatory target for cardiac sympathetic nerve activity (CSNA), affecting cardiac electrophysiology [25]. These features make TRPV1 an important research target in cardiovascular-related fields, providing critical insight into the pathological progression of cardiovascular diseases and informing future therapeutic strategies (Figure 1).

## 3. TRPV1 and Cardiovascular Disease

Cardiovascular diseases pose a serious threat to human health and remain a leading cause of morbidity and mortality worldwide. Clinically, these diseases are primarily classified as ischemic heart disease, cerebrovascular disease, valvular heart disease, cardiomyopathy, and heart failure. This review focuses on the regulatory role and molecular mechanisms of the TRPV1 channel in cardiovascular conditions such as myocardial infarction, ischemia–reperfusion injury, adverse cardiac remodeling, and heart failure. In addition, considering that hypertension and diabetes frequently involve significant cardiovascular dysfunction, this paper also summarizes the mechanisms by which TRPV1 may contribute to these diseases.

### 3.1. Myocardial Infarction and Myocardial Ischemia–Reperfusion Injury

Myocardial infarction (MI) refers to the ischemic necrosis of cardiomyocytes caused by obstruction of coronary blood flow. A primary therapeutic objective in modern cardiology is to develop strategies that minimize myocardial necrosis and optimize the subsequent repair process following MI. Percutaneous coronary intervention (PCI) is the most commonly used procedure to restore blood flow to the ischemic myocardium and reduce infarct size. Paradoxically, however, the restoration of blood flow can itself induce myocardial injury—a process known as myocardial ischemia–reperfusion injury (IRI) [26]. TRPV1 exerts multiple effects in the context of both MI and IRI. These include the release of neuropeptides that suppress inflammation via the TRPV1/CGRP/SP pathway, as well as the regulation of mitochondrial function and inhibition of apoptosis through the TRPV1/PI3K/Akt pathway (Table 1).

#### 3.1.1. Inflammation

The repair process following myocardial infarction is accompanied by a necessary inflammatory response that promotes healing and scar formation [27]. However, excessive inflammation can lead to adverse ventricular remodeling and deteriorating cardiac function. TRPV1 channels play a key regulatory role in modulating this response at both local and systemic levels. In MI mouse models, specific knockout of myocardial TRPV1 results in increased infiltration of neutrophils, macrophages, and myofibroblasts in the peri-infarct region, alongside significant upregulation of inflammatory factors and chemokines such as tumor necrosis factor-α (TNF-α), interleukin-6 (IL-6), IL-1β, monocyte chemoattractant protein-1 (MCP-1), and macrophage inflammatory protein-2 (MIP-2). Correspondingly, these mice exhibit larger infarct areas, increased collagen deposition, left ventricular dilation, reduced ejection fraction, and significantly decreased survival rates at 7 days post-MI [28]. These findings directly indicate that the normal function of TRPV1 is crucial for suppressing excessive inflammatory responses after myocardial infarction, thereby limiting myocardial injury and improving prognosis.

Furthermore, the cardioprotective effects of TRPV1 are mediated through multiple tiers of regulation. At the local level, effects are partially mediated through the release of sensory neuropeptides [6,29,30,31]. CGRP and SP are classic sensory neuropeptides secreted by sensory neurons. In vascular endothelial cells, CGRP and SP regulate vascular function through distinct mechanisms. Notably, exogenous CGRP supplementation reduced TNF-α and IL-6 levels and neutrophil infiltration in TRPV1-knockout MI mice. These results suggest that the TRPV1/CGRP/SP protective pathway may enhance tissue tolerance by suppressing local inflammation.

Beyond this local mechanism, recent research reveals that TRPV1-expressing vagal sensory neurons (VSNs) form a critical sensory node (Node 1) within a broader heart–brain neuroimmune loop [32]. Following MI, these neurons exhibit increased cardiac innervation. Their specific ablation not only attenuates local cardiac inflammation (reducing IL-1β and TNF-α) but also interrupts a maladaptive signal relay to the brain. This signal activates angiotensin II receptor type 1a (AT1aR)-expressing neurons in the hypothalamic paraventricular nucleus (PVN, Node 2), which in turn drives heightened sympathetic outflow and pro-inflammatory IL-1β signaling in the superior cervical ganglia (SCG, Node 3). Therefore, TRPV1 VSNs exert systemic anti-inflammatory and cardioprotective effects by preventing the activation of this central sympathetic excitatory and neuroinflammatory cascade. This integrated perspective positions TRPV1 as a key modulator at the interface of local tissue injury and systemic neuroimmune homeostasis post-MI.

#### 3.1.2. Cell Apoptosis

Myocardial ischemia induces apoptosis in cardiomyocytes, and reperfusion further exacerbates this process, ultimately leading to increased cardiomyocyte death. Consequently, inhibiting apoptosis is considered an effective strategy to mitigate ischemia–reperfusion injury [33].

Phosphatidylinositol 3-kinase (PI3K) is a lipid kinase that converts phosphatidylinositol 4,5-bisphosphate (PIP2) into phosphatidylinositol 3,4,5-trisphosphate (PIP3), which acts as a second messenger to activate downstream signaling cascades. Protein kinase B (Akt), a serine/threonine kinase activated by PIP3, plays a central role in promoting cell survival, inhibiting apoptosis, regulating the cell cycle, and enhancing metabolic activity. B-cell lymphoma 2 (Bcl-2) is an anti-apoptotic protein, whereas Bcl-2-associated X protein (Bax) is a pro-apoptotic homolog with opposing functions. The balance between Bcl-2 and Bax expression is a pivotal determinant of apoptosis regulation [34].

Experimental evidence indicates that, following ischemia–reperfusion injury, TRPV1 knockout mice exhibit a significantly larger myocardial infarct size and a higher proportion of TUNEL-positive cells compared to controls, accompanied by decreased Bcl-2/Bax expression and reduced phosphorylation levels of Akt and extracellular signal-regulated protein kinase 1/2 (ERK1/2). Administration of a PI3K inhibitor further increases myocardial infarct size and apoptosis in control mice, while suppressing levels of cardiac Bcl-2/Bax, Akt phosphorylation, and ERK1/2 phosphorylation. Notably, TRPV1 knockout mice do not display further impairment following PI3K inhibition. These findings suggest that TRPV1 mitigates cardiomyocyte apoptosis following ischemia–reperfusion injury by modulating Bcl-2/Bax via the PI3K/Akt signaling pathway [35].

Additionally, ERK1/2 activation plays an important role in cell survival and apoptosis regulation; however, the effects of PI3K inhibition on ERK1/2 activation may differ according to cell type, nature of the stimulus, and signal intensity [34,36]. Therefore, the precise contribution of the PI3K/ERK1/2 signaling pathway to TRPV1-mediated cardioprotection warrants further investigation.

#### 3.1.3. Mitochondrial Function

Mitochondrial dysfunction is one of the core pathological mechanisms underlying myocardial ischemia–reperfusion injury, typically characterized by dissipation of mitochondrial membrane potential, excessive reactive oxygen species (ROS) production, and impaired mitochondrial biogenesis. As a critical calcium channel, TRPV1 serves as a key regulator in these processes. However, its precise role within cardiomyocytes remains controversial, as its effects are highly context-dependent and subject to various regulatory mechanisms.

On one hand, *in vitro* cell models that are free from neuromodulatory interference have demonstrated that direct, potent activation of endogenous TRPV1 in cardiomyocytes exerts clearly detrimental effects on mitochondrial function. Studies show that treating H9C2 cells or primary cardiomyocytes with the TRPV1-specific agonist capsaicin exacerbates hypoxia/reoxygenation-induced intracellular calcium overload in a dose-dependent manner [14,36]. Such calcium overload directly triggers comprehensive mitochondrial dysfunction, manifested as marked reductions in mitochondrial membrane potential, increased superoxide anion generation, and downregulation of the ATP synthase β subunit—an essential marker of mitochondrial biosynthetic capacity. These alterations collectively result in decreased cellular viability and increased apoptosis. Conversely, TRPV1 inhibition by either the antagonist capsazepine or siRNA-mediated knockdown effectively alleviates these damages. Additionally, when the effects of neuropeptides are pharmacologically inhibited using CGRP and substance P receptor antagonists, TRPV1 knockout mouse hearts display significant improvements in LVDP, left ventricular systolic pressure (LVSP), and +LV dp/dt_max following ischemia/reperfusion [36]. These findings indicate that, once neural protective mechanisms are removed, direct activation of TRPV1 in cardiomyocytes induces calcium overload and mitochondrial dysfunction, thus promoting apoptosis during ischemia–reperfusion injury.

On the other hand, studies on *in vivo* animal models or at the level of intact organs have shown that TRPV1 activation can produce protective effects under certain regulatory circumstances, highlighting the context dependency of its actions. For example, low concentrations of capsaicin (10^−6^ mol/L or 0.1–1.0 mg/kg) elicit mild mitochondrial membrane potential loss but significantly reduce infarct size in whole-animal models—an effect abolished by TRPV1 antagonists. This paradox underscores the importance of the “degree” and “mode” of TRPV1 activation. Subsequent investigations have led to the hypothesis that TRPV1 may localize to mitochondria within cardiomyocytes, where its function could be critically regulated via interaction with the phosphatase calcineurin. This model is primarily based on observations of TRPV1 colocalization with mitochondrial markers and the functional effects of modulating the TRPV1-calcineurin interaction [14]. Biochemical detection of TRPV1 in isolated mitochondrial fractions provides supportive evidence [15]. Consistent with this regulatory model, the use of a synthetic peptide (V1-cal) designed to specifically disrupt the TRPV1–calcineurin interaction allowed for precise modulation of TRPV1 activity during reperfusion without direct channel blockade. This intervention conferred pronounced cardioprotective effects both *in vitro* and *in vivo*, stabilizing mitochondrial membrane potential and significantly reducing infarct size [14]. These findings suggest that, if the mitochondrial localization hypothesis holds true, targeting the TRPV1-calcineurin interface could be a novel strategy to mitigate reperfusion injury by preserving mitochondrial integrity.

In summary, TRPV1 exerts dual effects on mitochondrial function and cell fate during myocardial ischemia–reperfusion injury. At the molecular level, mitochondrial TRPV1 activation directly mediates mitochondrial dysfunction under hypoxic/reoxygenation stress. However, *in vivo* models and targeted therapeutic interventions demonstrate that precise modulation of TRPV1-calcineurin interactions can transform TRPV1 into a promising cardioprotective target. Therefore, a thorough understanding of TRPV1’s role in ischemia–reperfusion injury necessitates careful distinction between its direct cellular effects and its integrated regulatory potential within the intact physiological system.

**Table 1:** The expression of TRPV1 in the cardiovascular system and its impact on ischemic heart disease.

References	Localization	TRPV1 Action	Experimental Model and Modeling Diseases	Impact	Pathway
Huang et al. [28]	Sensory afferent fibers innervating the heart	Inhibition	Male WT and TRPV1 knockout mice; MI	Inhibit inflammation	/
Sexton et al. [6]	Sensory C-fibers	Activation	Male Wistar rats and TRPV1 knockout mice; IRI	Inhibit inflammation	TRPV1/CGRP/SP
Zhong et al. [30]	Heart	Activation	Male WT and TRPV1 gene knockout mice; IRI	Inhibit inflammation	TRPV1/CGRP/SP
Zhong et al. [31]	Heart	Activation	Male WT and gene-targeted TRPV1-null mutant mice; IRI	Inhibit inflammation	TRPV1/CGRP/SP
Yadav et al. [32]	VSNs	Inhibition	TRPV1-Cre mice; MI	Inhibit inflammation Modulate sympathetic responses	/
Jiang et al. [35]	Heart	Activation	Male WT and TRPV1 knockout mice; IRI	Reduce apoptosis	TRPV1/PI3K/Akt
Hurt et al. [14]	Mitochondria in cardiomyocytes	Moderate activation	Male Sprague-Dawley rats and TRPV1 knockout rats; PNCMs; IRI	Regulate mitochondrial function	/
Sun et al. [36]	Cardiomyocytes	Activation	Male WT and TRPV1 knockout mice, H9C2 cells; H/R injury and IRI	Regulate mitochondrial function	/

TRPV1: transient receptor potential vanilloid 1; WT: wild-type; MI: myocardial infarction; VSNs: vagal sensory neurons; IRI: ischemia–reperfusion injury; H/R: hypoxia/reoxygenation; SP: substance P; CGRP: calcitonin gene-related peptide; PI3K: phosphatidylinositol 3-kinase; Akt: protein kinase B.

### 3.2. Cardiac Pathological Remodeling

The heart is capable of adaptive hypertrophy in response to physiological stimuli such as exercise. This reversible compensatory mechanism increases cardiac output by moderately enhancing myocardial mass, thereby meeting elevated metabolic demands for oxygen and nutrients. However, under pathological conditions—including excessive neurohumoral activation, myocardial injury, and pressure overload—this adaptive capacity is disrupted, leading the heart to transition from compensatory hypertrophy to pathological remodeling [2,37].

A central feature of this process is myocardial interstitial fibrosis, which is primarily manifested as abnormal deposition of extracellular matrix (ECM) within the myocardial interstitium. This results in expansion of the endomysial and perimysial spaces, further activating fibroblasts and promoting their differentiation into myofibroblasts capable of synthesizing collagen. Such cellular and molecular events drive the initiation and progression of myocardial fibrosis [38], ultimately contributing to left ventricular enlargement and compromised systolic and diastolic function—a syndrome known as cardiac pathological remodeling [2].

TRPV1 channels play a complex and pivotal role throughout this process. On one hand, TRPV1 counteracts cardiac injury by improving mitochondrial function, suppressing the pro-fibrotic TGF-β1/Smad2/3 signaling pathway, and activating the protective eNOS/NO pathway. On the other hand, calcium signaling mediated by TRPV1 may promote myocardial hypertrophy under certain pathological conditions. This apparent contradiction arises from differences in pathological modeling approaches and the diverse signaling networks activated by TRPV1 in various cell types. TRPV1’s effects span multiple levels—including mitochondrial function, intracellular signaling, and neuro–immune interactions—rendering its function highly context-dependent (Table 2).

#### 3.2.1. Cell Signaling Pathways

Beyond regulating organelle function, TRPV1 also acts as a central molecular node in pathological cardiac remodeling by modulating two core intracellular signaling pathways, thereby achieving bidirectional protection of cardiac structure and function. Specifically, TRPV1 simultaneously inhibits the pro-fibrotic TGF-β/Smad signaling pathway while activating the protective eNOS/NO/cGMP signaling cascade.

Transforming growth factor (TGF)-β1 and its downstream Smad2/3 pathway serve as principal regulators of extracellular matrix (ECM) synthesis and tissue fibrosis. In pressure overload-induced cardiac remodeling, this axis is abnormally activated, leading to collagen deposition and myocardial fibrosis. Experimental studies show that in a mouse model of abdominal aortic banding (AB)-induced cardiac remodeling, dietary supplementation with 0.01% capsaicin significantly attenuates pressure-induced cardiac hypertrophy and dysfunction, and effectively suppresses excessive deposition of collagen I, collagen III, and fibronectin in both the myocardial interstitium and perivascular regions [39]. At the molecular level, capsaicin treatment results in pronounced downregulation of TGF-β1 and connective tissue growth factor (CTGF) expression, inhibition of Smad2/3 phosphorylation, and modulation of matrix metalloproteinase (MMP-2, MMP-9, MMP-13) expression, thereby alleviating ECM accumulation. Importantly, these protective effects are completely abolished in TRPV1 knockout mice, indicating that the cardioprotective actions of capsaicin are strictly dependent on TRPV1. Thus, dietary capsaicin may combat stress-induced cardiac remodeling by activating TRPV1, inhibiting the TGF-β1/Smad2/3 signaling pathway, and regulating MMP expression.

Endothelial nitric oxide synthase (eNOS), predominantly expressed in endothelial cells, catalyzes the conversion of L-arginine to NO. The eNOS/NO signaling pathway is crucial for maintaining cardiovascular homeostasis, and its activation protects against myocardial injury in various pathological contexts. For example, in β-adrenergic overstimulation models, eNOS overexpression reduces cardiac hypertrophy and heart weight/body weight (HW/BW) ratio by enhancing NO production [40]. Given TRPV1’s function as a calcium-permeable channel capable of modulating intracellular Ca^2+^ dynamics, and acknowledging that the Ca^2+^/calmodulin system is the primary upstream regulator of eNOS activity, researchers investigated whether TRPV1 contributes to cardioprotection via this pathway [41]. In an isoproterenol-induced myocardial fibrosis model, TRPV1 transgenic overexpression led to significant improvement in HW/BW, left ventricular weight/body weight ratio (LVW/BW), left ventricular end-diastolic pressure (LVEDP), myocardial fibrosis area, and total collagen content. Mechanistically, TRPV1 overexpression enhanced Akt and eNOS phosphorylation, leading to increased NO and cyclic guanosine monophosphate (cGMP) production. Conversely, administration of the eNOS inhibitor L-NAME reversed the anti-fibrotic effects conferred by TRPV1 overexpression. These results suggest that TRPV1 may suppress cardiac remodeling secondary to sympathetic overactivation by promoting Ca^2+^ influx and activating the Akt/eNOS/NO/cGMP signaling axis, thereby inhibiting myocardial fibroblast proliferation and collagen accumulation.

Taken together, TRPV1 exerts its protective effects in cardiac fibrosis via distinct downstream signaling pathways, depending on pathogenic context. In pressure overload models, TRPV1 activation suppresses the canonical pro-fibrotic TGF-β/Smad pathway, fundamentally minimizing abnormal ECM synthesis. In the setting of sympathetic overactivation, TRPV1 activation promotes eNOS/NO/cGMP signaling, enhances cardiovascular homeostasis, and inhibits fibroblast activation. Together, these seemingly independent pathways underscore TRPV1’s role as a multifunctional molecular node in the cardiac stress response. Notably, cGMP and its downstream effector, protein kinase G, have been demonstrated to directly inhibit Smad3 transcriptional activity by phosphorylating specific serine/threonine residues (Ser309/Thr388), thereby preventing Smad3 nuclear translocation [42]. This finding suggests that cGMP may serve as an intrinsic hub connecting the two pathways, ultimately integrating TRPV1 activation into a synergistic anti-fibrotic signaling network.

#### 3.2.2. Mitochondrial Function

Mitochondrial dysfunction is a core mechanism driving pathological cardiac remodeling, characterized by impaired mitochondrial quality control, diminished energy metabolism reserves, and increased oxidative stress, which together activate pro-apoptotic signaling pathways. Recent evidence suggests that TRPV1 may participate in maintaining mitochondrial quality and function during pathological cardiac remodeling. Its presumed protective role has been supported by multiple experimental observations [8,15,43], but its subcellular mechanisms—such as precise subcellular localization—require more comprehensive evidence to substantiate [14,15,44].

Experimental models suggest a functional link between TRPV1 activity and the regulation of mitochondrial calcium homeostasis and membrane potential. Ca^2+^ is crucial for the regulation of apoptosis, hypertrophy-related gene expression, and myocardial fibrosis [45]. Overactivation of the endothelin system, for instance, can provoke Ca^2+^ overload in cardiomyocytes, triggering maladaptive hypertrophy and fibrosis. In models of cold-induced or endothelin-1 (ET-1)-induced cardiac stress, TRPV1 expression is downregulated by glycogen synthase kinase-3β (GSK3β), and recovery of its activity is critical for improving mitochondrial dysfunction [43]. Specifically, cold stress inhibits Akt phosphorylation and activates GSK3β, resulting in reduced expression of both TRPV1 and peroxisome proliferator-activated receptor-γ coactivator-1α (PGC-1α), while simultaneously increasing uncoupling protein 2 (UCP2) expression. Restoration of TRPV1 function—via cardiac-specific ETA receptor knockout (ETAKO) or exogenous agonists—can reverse cold stress-induced declines in mitochondrial membrane potential (MMP), mitigate excessive ROS production, and normalize intracellular calcium handling (reflected by restored calcium transients and improved calcium clearance after electrical stimulation). In contrast, TRPV1 antagonism reproduces the injurious phenotype, supporting the notion that TRPV1 is a critical effector in protecting against mitochondrial dysfunction and pathological remodeling.

Secondly, studies, especially those employing chronic stress models such as a high-salt diet, suggest that TRPV1 activation protects mitochondrial energy metabolism and protein homeostasis—effects that are potentially mediated by modulation of the sirtuin 3 (SIRT3)–complex I (CI) axis and by regulation of SIRT3 activity and mitochondrial CI function, thereby conferring cardioprotection. CI dysfunction leads to NADH accumulation, reducing the NAD^+^/NADH ratio, which in turn impairs SIRT3 activity. This results in hyperacetylation of mitochondrial proteins (e.g., CI subunit NDUFA9), thereby increasing sensitivity to mitochondrial permeability transition pore (mPTP) opening and cell death [46]. In high-salt diet-induced cardiac injury models, capsaicin-mediated TRPV1 activation significantly enhances mitochondrial CI function, increases ATP production, and upregulates both SIRT3 and NDUFA9 expression. These benefits are lost in TRPV1 knockout mice or after TRPV1 antagonism, indicating that TRPV1 activity is indispensable for SIRT3-CI axis integrity and mitochondrial protein homeostasis [15]. These findings support a model in which TRPV1 activity is crucial for maintaining the SIRT3–CI axis, thereby promoting myocardial resistance to metabolic stress.

Furthermore, TRPV1 alleviates oxidative stress-induced cardiac injury by reinforcing mitochondrial antioxidant defenses. Peroxisome proliferator-activated receptor-δ (PPAR-δ), a member of the nuclear receptor superfamily, governs energy metabolism and antioxidant responses. Pathologically, inducible nitric oxide synthase (iNOS) is upregulated, resulting in overproduction of NO that reacts with superoxide to generate strong oxidants such as peroxynitrite, driving protein nitrosative damage (marked by elevated 3-nitrotyrosine) and exacerbating oxidative–nitrosative stress. UCP2, an intrinsic mitochondrial antioxidant protein, reduces membrane potential and limits free radical generation [47]. In high-salt-induced cardiac remodeling, long-term dietary capsaicin significantly attenuates increases in the HW/BW ratio, reduces left ventricular chamber enlargement, improves fractional shortening (FS), and lessens myocardial collagen deposition [8]. Such protective effects are absent in TRPV1 knockout mice, highlighting their dependence on TRPV1. Mechanistically, TRPV1 activation markedly upregulates PPAR-δ and UCP2 expression while reducing iNOS and 3-nitrotyrosine levels, implying that TRPV1 coordinates the mitochondrial antioxidant response and mitigates nitrosative stress.

Collectively, experimental data from various stress models correlate TRPV1 activity with improved mitochondrial parameters and attenuated pathological remodeling. This has led to a conceptual framework wherein TRPV1 is seen as orchestrating mitochondrial protection through the aforementioned mechanisms. In acute stress models such as cold exposure or ET-1 stimulation, TRPV1, acting as a downstream mediator of GSK3β, restores membrane potential, mitigates oxidative stress, and normalizes calcium homeostasis. Under chronic stress (e.g., high-salt diet), TRPV1 activates the SIRT3–CI axis and PPAR-δ/UCP2 antioxidant pathway to preserve mitochondrial energy metabolism and redox balance. Collectively, these actions suppress myocardial hypertrophy, fibrosis, and contractile dysfunction, highlighting the potential of targeting TRPV1-related pathways for intervention in pathological cardiac remodeling.

It should be emphasized that although multiple studies have suggested the influence of TRPV1 on mitochondrial function, its precise site of action—whether it directly localizes to the mitochondrial membrane or indirectly transmits signals through other organelles—remains unclear. A recent study in rat cardiomyoblast H9c2 cells, employing subcellular fractionation, immunoblotting, immunofluorescence colocalization, proximity ligation assays, and electrophysiological recordings, demonstrated that TRPV1 is primarily localized to the endoplasmic reticulum (ER) membrane and enriched at mitochondria-associated ER membranes (MAMs)—the physical contact sites between ER and mitochondria. Functional calcium imaging using compartment-specific probes revealed that TRPV1 activation triggers ER calcium release that is rapidly taken up by adjacent mitochondria [44]. This finding suggests that TRPV1, rather than being a mitochondrial channel, may regulate mitochondrial function indirectly by mediating ER-mitochondrial calcium transfer at MAMs. This notion is consistent with earlier functional evidence demonstrating, in both transverse aortic constriction-induced mouse models and phenylephrine-treated neonatal rat cardiomyocytes, that TRPV1 activation promotes MAM formation via the AMP-activated protein kinase (AMPK)-mitofusin (MFN)2 pathway, thereby counteracting pressure overload-induced cardiac hypertrophy and improving MMP, enhancing ATP production, and reducing mitochondrial ROS. Notably, disruption of MAMs by MFN2 siRNA abolished TRPV1-mediated mitochondrial protection, confirming that MAM integrity is essential for TRPV1’s cardioprotective effects [48]. However, as these studies were conducted in an immature cardiac precursor cell line or neonatal cardiomyocytes, whether these conclusions can be fully extrapolated to functionally complex adult cardiomyocytes requires future validation in more mature models.

Taken together, although the exact subcellular localization of TRPV1 in adult cardiomyocytes awaits definitive confirmation, the convergence of structural localization data and functional evidence has definitively established that TRPV1 modulates cardiac remodeling by regulating mitochondrial function. This highlights the potential for targeting TRPV1-related pathways to intervene in pathological cardiac remodeling. However, the precise site and mechanism by which TRPV1 influences cardiac mitochondrial function require further investigation in subsequent studies.

#### 3.2.3. Calcium Signaling and Neuro–Immune Regulation

In pathological cardiac remodeling, a complex network involving calcium signaling and neuro–immune interactions plays a central role. As a calcium-permeable channel, TRPV1 activation induces calcium influx, which subsequently drives transcriptional programs associated with myocardial hypertrophy, fibrosis, and apoptosis through downstream signaling pathways such as the calcium/calmodulin-dependent kinase IIδ (CaMKIIδ)–mitogen-activated protein kinase (MAPK) cascade and intracellular polyamine metabolism [9,49].

Experimental evidence shows that in the transverse aortic constriction (TAC) model, mice lacking functional TRPV1 develop less severe cardiac remodeling: compared with wild-type mice, they exhibit significantly attenuated increases in the heart weight/body weight (HW/BW) ratio, cardiomyocyte cross-sectional area, and markers of fibrosis and apoptosis. Capsaicin (CAP) and the endogenous TRPV1 agonist anandamide (ANA) increase intracellular Ca^2+^ by activating TRPV1, thereby markedly upregulating phosphorylation of CaMKIIδ, extracellular signal-regulated kinase (ERK), and p38 MAPK. This cascade leads to elevated expression of ornithine decarboxylase (ODC), enhanced polyamine synthesis, and a pronounced increase in cardiomyocyte cross-sectional area. *In vivo* experiments further confirm that TAC-induced upregulation of TRPV1 occurs concomitantly with activation of this pro-hypertrophic kinase network, while the TRPV1 antagonist capsazepine suppresses this signaling and improves cardiac structure and function [9]. Earlier pharmacological studies support this view. The TRPV1 antagonist BCTC effectively ameliorates ventricular dilation and improves ejection fraction in TAC-treated mice, while reducing expression levels of atrial natriuretic peptide, brain natriuretic peptide, collagen (*Col3a1*), MMP-9, IL-6, and TGF-β receptor 1, as well as markedly decreasing the pro-apoptotic protein cleaved caspase-3 [49]. Collectively, these studies delineate a remodeling-promoting axis initiated by TRPV1-mediated Ca^2+^ influx and a network of downstream protein kinases.

Conversely, another study revealed a distinct neuro-immunoregulatory module within this complex regulatory network. In this context, TRPV1 knockout mice subjected to TAC surgery exhibited more pronounced cardiac hypertrophy, ventricular wall thickening, loss of systolic function, and myocardial fibrosis compared to wild-type mice. These changes were accompanied by more severe inflammation, as evidenced by elevated TNF-α and IL-6 levels and increased macrophage infiltration. Mechanistically, pressure overload triggers upregulation of TRPV1 in the hearts of wild-type mice and promotes the release of CGRP—an effect absent in TRPV1 knockout mice. These results indicate that functional TRPV1 in sensory neurons can mediate neuropeptide release, such as CGRP, thereby limiting inflammation and conferring compensation against adverse remodeling and functional deterioration [50].

In summary, research into pressure overload-induced cardiac remodeling indicates that TRPV1 channel activation exerts seemingly contradictory effects, suggesting the presence of a more integrated neuro-immune–calcium signaling regulatory network. Existing evidence supports at least two distinct core modules: First, in non-neuronal cells (e.g., cardiomyocytes), TRPV1 activation triggers Ca^2+^ influx and subsequently initiates a cascade in the CaMKIIδ/MAPK network, driving classic pathophysiological processes such as hypertrophy, fibrosis, and apoptosis. Second, under chronic stress, upregulation of TRPV1 in sensory nerves promotes the release of neuropeptides like CGRP, inhibiting macrophage infiltration and pro-inflammatory cytokine production, and thus exerts anti-inflammatory and compensatory protective effects.

The apparent discrepancies between different studies are therefore not mutually exclusive. Rather, they likely reflect that the dominance of “calcium signaling-driven” versus “neuro–immune regulation” modules varies according to experimental conditions, disease stage, and intervention strategy. Systemic approaches such as gene knockout or TRPV1 antagonism can concurrently disrupt the balance of these modules, ultimately manifesting as divergent or even opposing phenotypes. Future research should aim to precisely map activation patterns and interaction dynamics between these regulatory modules. Such insights will be essential for fully elucidating the context-dependent roles of TRPV1 and for informing the development of precise therapeutic strategies for pathological cardiac remodeling.

**Table 2:** The expression of TRPV1 in the cardiovascular system and its impact on adverse cardiac remodeling.

References	Localization	TRPV1 Action	Experimental Model and Modeling Diseases	Impact	Pathway
Wang et al. [39]	Heart	Activation	Male WT and TRPV1 knockout mice; Cardiac hypertrophy and fibrosis	Inhibit fibrotic signaling	TRPV1/TGF-β1/Smad2/3
Wang et al. [41]	Myocardium	Activation	Male WT mice and transgenic mice overexpressing TRPV1; Myocardial fibrosis	Promote NO-mediated protection	TRPV1/eNOS/NO/cGMP
Zhang et al. [43]	Heart	Activation	Male WT and ETA receptor knockout mice; Cardiac hypertrophy	Preserve MMP	/
Lang et al. [15]	Both in the mitochondria and cytoplasm of cardio-myoblasts	Activation	WT and TRPV1 knockout mice; Cardiac hypertrophy	Improve mitochondrial CI function	TRPV1/SIRT3
Gao et al. [8]	Cardiomyocytes	Activation	Male WT and TRPV1-null mice; Cardiac hypertrophy and fibrosis	Reduce oxidative/nitrosative stress	TRPV1/PPAR-δ/UCP2
Wang et al. [48]	Cardiomyocytes	Activation	Male WT mice and neonatal rat cardiomyocytes; Cardiac hypertrophy	Improve mitochondrial morphology and preserve MMP	TRPV1/AMPK/MFN2/MAM formation
Chen et al. [9]	Heart	Inhibition	Male WT mice and neonatal rat cardiomyocytes; Cardiac hypertrophy	Inhibit calcium overload	CaMKIIδ–MAPK–polyamine pathways
Horton et al. [49]	Heart	Inhibition	Male WT mice; Cardiac hypertrophy	Inhibit calcium overload	Inhibit TRPV1/CaMKIIδ/ MAPK
Zhong et al. [50]	Heart	Inhibition	Male WT and TRPV1 gene knockout mice; Cardiac hypertrophy	Exacerbate inflammation	Inhibit TRPV1/CGRP

TRPV1: transient receptor potential vanilloid 1; WT: wild-type; ETA: endothelin receptor A; eNOS: endothelial nitric oxide synthases; NO: nitric oxide; TGF-β1: transforming growth factor β1; MMP: mitochondrial membrane potential; SIRT3: Sirtuin 3; CaMKIIδ: calmodulin-dependent protein kinase Iiδ; MAPK: mitogen-activated protein kinase; AMPK: AMP-activated protein kinase; MFN2: mitofusin 2; MAM: mitochondria-associated endoplasmic reticulum membrane.

### 3.3. Heart Failure

Heart failure (HF) is a complex clinical syndrome arising from ventricular systolic or diastolic dysfunction, typically triggered by structural or functional cardiac abnormalities. Recent research indicates that excessive activation of the cardiac sympathetic afferent reflex (CSAR) plays a central role in the pathogenesis and progression of HF. TRPV1, predominantly expressed on cardiac afferent nerve terminals, serves as a key molecular target for CSAR regulation. Selective ablation of TRPV1-positive afferent nerves using resiniferatoxin (RTX) has been shown to effectively suppress CSAR, representing a novel therapeutic approach for HF (Table 3).

Experimental studies have demonstrated that applying RTX (50 μg/mL) to the cardiac epicardium of rats with chronic heart failure (CHF) selectively eliminates TRPV1-expressing sympathetic afferent fibers [51]. Compared to controls, RTX-treated CHF rats displayed significant reductions in LVEDP and increased minimum rate of pressure change (dP/dtmin), indicating improved cardiac diastolic function. Furthermore, under stimulation with the β-adrenergic receptor agonist isoproterenol, RTX enhanced the maximum rate of pressure change (dP/dtmax), signifying enhanced myocardial contractile reserve. Pressure-volume analysis showed that RTX decreased the slope of the end-diastolic pressure–volume relationship, improving ventricular compliance. At the molecular level, RTX treatment significantly decreased myocardial fibrosis, reduced levels of cleaved caspase-3, and lowered the proportion of TUNEL-positive apoptotic cells, suggesting that TRPV1 afferent ablation can inhibit cardiomyocyte apoptosis and mitigate adverse cardiac remodeling in CHF rats. Similar findings were observed in a TAC rat model [52]. Here, intrathecal injection of RTX (2 μg/10 μL) was used to block the central projections of TRPV1-positive cardiac afferent neurons in the spinal dorsal horn, thereby reducing the hyperactivated cardiac sympathetic nerve activity (CSNA). RTX intervention decreased expression of the pro-apoptotic protein Bax, increased the anti-apoptotic protein Bcl-2, and inhibited caspase-3 activation, further confirming that TRPV1 ablation can suppress cardiomyocyte apoptosis, improving ventricular compliance and diastolic function.

Beyond structural and functional improvements, TRPV1 afferent ablation also provides protective effects against the electrophysiological disturbances characteristic of HF. These disturbances—including prolonged action potential duration (APD), aberrant calcium handling, and repolarization instability—are major contributors to the heightened risk of malignant ventricular arrhythmias in HF. Studies have shown that intrathecal RTX in HF rats effectively suppresses CSNA, decreases plasma norepinephrine levels, shortens APD, reduces APD alternans susceptibility, and lowers the incidence of programmed electrical stimulation-induced ventricular tachycardia/fibrillation [25]. Mechanistically, RTX reversed excessive phosphorylation of CaMKII and ryanodine receptor 2 (RyR2) in failing myocardium, implicating regulatory effects on calcium-handling protein stability and arrhythmia mitigation. These protective effects have also been validated in large animal models. In a porcine myocardial infarction model, RTX treatment significantly reduced electrically induced ventricular tachycardia/fibrillation incidence and arrhythmia indices, attenuated infarct zone fibrosis, improved connexin 43 distribution, and enhanced electrical conduction homogeneity. Additionally, RTX ameliorated post-infarction adverse remodeling, including preservation of left ventricular ejection fraction (LVEF), reductions in left ventricular end-systolic volume (LVESV) and end-diastolic diameter (LVEDD), and prevention of ventricular wall thinning [53].

Collectively, these findings demonstrate that selective ablation of TRPV1-positive cardiac afferent nerves—via peripheral or central approaches—can effectively attenuate sympathetic excitation and reduce intracellular calcium overload in cardiomyocytes. This confers multiple cardioprotective benefits in heart failure, including significant improvement of cardiac systolic and diastolic function, enhancement of ventricular compliance, suppression of arrhythmic risk, inhibition of myocardial hypertrophy and fibrosis, and reduction in cardiomyocyte apoptosis. The underlying mechanisms involve comprehensive regulation of sympathetic nerve activity, stabilization of calcium-handling proteins, and modulation of apoptotic signaling pathways. These insights provide compelling experimental and translational evidence for neuromodulatory therapies targeting TRPV1 in heart failure.

**Table 3:** The expression of TRPV1 in the cardiovascular system and its impact on heart failure.

References	Localization	TRPV1 Action	Experimental Model and Modeling Diseases	Impact	Pathway
Wang et al. [51]	Cardiac sympathetic afferent fibers	Inhibition	Male Sprague–Dawley rats; CHF	Inhibit sympathetic excitability	/
Wang et al. [52]	Left ventricular and T1–T4 spinal	Inhibition	Male Sprague–Dawley rats; HF	Inhibit sympathetic excitability	/
Wu et al. [25]	Left ventricular and T1–T2 spinal	Inhibition	Male Sprague–Dawley rats; HF	Inhibit sympathetic excitability	Inhibit TRPV1/CaMKII
Yoshie et al. [53]	cardiomyocytes	Inhibition	Yorkshire pigs with normal hearts and chronic anterior MI; MI and VAs	Inhibit sympathetic excitability	/

TRPV1: transient receptor potential vanilloid 1; CHF: chronic heart failure; HF: heart failure; MI: myocardial infarction; VAs: ventricular arrhythmogenesis.

### 3.4. Risk Factors for Cardiovascular Dysfunction: Hypertension and Diabetes

Hypertension and diabetes are two predominant risk factors for the development of cardiovascular dysfunction. Chronic hypertension elevates vascular wall shear stress, resulting in endothelial dysfunction and subsequent hemodynamic injury. Persistent elevation of cardiac afterload further induces maladaptive left ventricular remodeling, thereby increasing the risk of heart failure. Meanwhile, diabetes exacerbates inflammatory responses and oxidative stress within the cardiovascular system. Insulin resistance, a hallmark of diabetes, leads to reduced bioavailability of NO and impaired endothelium-dependent vasodilation, further compromising vascular function.

The following sections will focus on the regulatory roles of TRPV1 and its associated molecular mechanisms in the context of hypertension, diabetes, and resulting cardiovascular impairment, providing insights into potential therapeutic strategies targeting TRPV1 for mitigating these high-risk factors.

#### 3.4.1. Hypertension

Hypertension is a chronic disease characterized by persistently elevated arterial blood pressure, defined as a systolic blood pressure of ≥140 mmHg and/or a diastolic blood pressure of ≥90 mmHg at rest. As one of the most prevalent chronic diseases worldwide, hypertension affects approximately 33% of adults aged 30–79 globally and is a major risk factor for cardiovascular events such as stroke and coronary heart disease [22,54]. Endothelial dysfunction is a central component of vascular injury in hypertension, with oxidative stress and chronic inflammation serving as primary drivers. TRPV1 may exert a protective role in hypertension-induced cardiovascular dysfunction by regulating these processes at both the peripheral vascular and central nervous system levels (Table 4).

Endothelial function depends on the bioavailability of NO, while oxidative stress leads to excessive ROS production, which is one of the primary causes of reduced NO levels and endothelial dysfunction [55]. Activation of TRPV1 has been demonstrated to suppress oxidative stress and improve endothelial dysfunction in experimental animals with hypertension or metabolic abnormalities via the PKA/eNOS/NO signaling axis. Long-term capsaicin intake enhances endothelium-dependent vasodilation and reduces blood pressure (including nocturnal blood pressure) in spontaneous hypertension and high-salt diet hypertension models by activating TRPV1, increasing PKA and eNOS phosphorylation levels in mesenteric arteries, promoting NO production, and reducing ROS. This effect is absent in TRPV1 knockout mice, confirming its dependence [22,56]. Beyond directly regulating vascular tone, activation of TRPV1 exerts multiple protective effects on hypertensive hearts. In L-nitro-arginine methyl ester (L-NAME)-induced hypertensive rat models, researchers observed that subcutaneous injection of capsaicin significantly reduced mean arterial pressure (MAP) while improving cardiac mechanical function and coronary vascular resistance. At the molecular level, capsaicin concurrently elevated myocardial eNOS and guanosine triphosphate cyclohydrolase 1 (GTPCH-1) expression, increased tetrahydrobiopterin (BH4) and NO levels, enhanced cGMP, and reduced phosphodiesterase-3 (PDE-3) activity. Concurrently, total antioxidant capacity increased while malondialdehyde (MDA) levels decreased [57]. These findings collectively reveal that TRPV1 activation may reduce oxidative stress and improve endothelial dysfunction through the eNOS/NO/cGMP pathway, thereby synergistically protecting vascular and cardiac function.

TRPV1 also plays a crucial role in regulating hypertension-related inflammatory responses through mechanisms involving direct cellular actions, systemic peripheral regulation, and potentially central neuromodulation. At the cellular level, TRPV1 exerts anti-inflammatory effects via a Ca^2+^-dependent dual pathway. Nuclear factor kappa-B (NF-κB) is a primary inflammatory signaling pathway; its excessive activation drives the expression of pro-inflammatory factors [58]. Research indicates that TRPV1 activation not only increases NO production via the PI3K/Akt/eNOS/NO signaling axis but also directly suppresses NF-κB activation, thereby reducing endothelial cell inflammation [59,60]. Treatment of endothelial cells with TRPV1 agonists significantly increases eNOS phosphorylation and NO production, and this effect is blocked by TRPV1 antagonists, siRNA, PI3K/Akt/CaMKII inhibitors, or the calcium chelator EGTA [59]. Notably, immunoprecipitation experiments revealed that TRPV1 binding to eNOS, Akt, and CaMKII significantly increased following TRPV1 activation. This suggests that TRPV1 not only regulates eNOS activity through its channel function but may also act as a scaffold protein to promote interactions among eNOS, Akt, and CaMKII. Researchers further demonstrated in human umbilical vein endothelial cells and a salt-sensitive hypertension mouse model that capsaicin pretreatment increases cellular NO production in a dose-dependent manner via the aforementioned mechanism. It also inhibits LPS-induced inflammatory factor production, adhesion molecule expression, and NF-κB activity, thereby exerting anti-inflammatory and cardioprotective effects [60].

At the systemic level, TRPV1 plays a role in regulating metabolic inflammation and sympathetic nervous system activity. Dysregulation of these TRPV1-mediated pathways is closely associated with the development and progression of conditions such as obesity, hypertension, and renal insufficiency. Research has shown that deletion of the TRPV1 gene aggravates Western diet (WD)-induced nocturnal hypertension and impairs baroreflex function [61]. Compared with wild-type mice, TRPV1 knockout mice fed a WD exhibited higher nocturnal MAP, elevated plasma leptin and adipose tissue inflammatory factors (IL-6, TNF-α), as well as impaired renal sympathetic regulation. This indicates that functional TRPV1 helps maintain circadian blood pressure regulation, at least in part, by suppressing leptin elevation and associated inflammatory factor secretion, thereby reducing sympathetic overactivity.

Expanding on the systemic and neural dimensions, recent evidence suggests that the cardiovascular benefits of TRPV1 activation may also involve actions within key central autonomic nuclei. The hypothalamic PVN is a critical hub for blood pressure regulation and sympathetic outflow. Intriguingly, chronic infusion of capsaicin directly into the PVN of SHRs significantly lowered blood pressure and improved cardiac hypertrophy. This was associated with increased expression of the deacetylase SIRT1, reduced oxidative stress, and suppressed NF-κB activity and pro-inflammatory cytokine levels within the PVN itself [62]. Although this study did not explicitly confirm TRPV1-mediated effects, given that capsaicin is a classic TRPV1 agonist, these findings imply a plausible mechanism: capsaicin may suppress excessive sympathetic excitation and lower blood pressure by activating TRPV1 in central cardiovascular regulatory regions such as the PVN, thereby alleviating local neuroinflammation and oxidative stress. This hypothesized pathway aligns with the concept emphasized in studies of MI: that TRPV1-expressing sensory pathways participate in cardio-cerebral communication and sympathetic regulation [32]. It must be explicitly noted, however, that this remains a hypothesis requiring further validation. Future studies should elucidate the precise mechanism of action through more in-depth investigations, such as TRPV1-specific knockout or antagonism within the PVN.

In summary, under hypertensive conditions, TRPV1 activation exerts cardiovascular protective effects through multiple targets. Regarding oxidative stress, its core mechanism lies in activating the TRPV1/PKA/eNOS signaling axis, elevating NO-cGMP levels, enhancing antioxidant defense, and improving endothelium-dependent vasodilation. Regarding inflammatory responses, it activates the PI3K/Akt/eNOS pathway via Ca^2+^ influx while suppressing NF-κB activity, and modulates leptin-mediated inflammation and sympathetic activity at the systemic level. Collectively, these mechanisms elucidate TRPV1’s potential for blood pressure regulation and target organ protection in salt-sensitive, metabolic, and neuroendocrine-related hypertension.

**Table 4:** The expression of TRPV1 in the cardiovascular system and its impact on hypertension.

References	Localization	TRPV1 Action	Experimental Model and Modeling Diseases	Impact	Pathway
Yang et al. [22]	ECs and mesenteric arteries	Activation	SHRs and Wistar rats; Hypertension	Inhibit oxidative stress	TRPV1/PKA/eNOS/NO
Hao et al. [56]	Mesenteric resistance arteries	Activation	Male WT and TRPV1 null mice; Hypertension	Inhibit oxidative stress	TRPV1/PKA/eNOS/NO
Torres-Narváez et al. [57]	ventricular tissue	Activation	Male Wistar rats; SAHT	Inhibit oxidative stress	TRPV1/eNOS/NO/cGMP
Wang et al. [60]	ECs	Activation	Renal MVECs from salt-sensitive hypertensive mice and HUVECs; Hypertension	Inhibit inflammation	TRPV1/Ca^2+^/PI3K/Akt/eNOS/NO pathway and inhibit NF-κB
Zhong et al. [61]	Renal nerves	Inhibition	Male WT and TRPV1 gene knockout mice; Hypertension	Exacerbate inflammation	/

TRPV1: transient receptor potential vanilloid 1; WT: wild-type; ECs: endothelial cells; SHRs; spontaneously hypertensive rats; PKA: protein kinase A; eNOS: endothelial nitric oxide synthases; NO: nitric oxide; SAHT: systemic arterial hypertension; cGMP: cyclic adenosine monophosphate; MVECs: microvascular endothelial cells; HUVECs: human umbilical vein endothelial cells; PI3K: phosphatidylinositol 3-kinase; Akt: protein kinase B; NF-κB: nuclear transcription factor-κB.

#### 3.4.2. Diabetes

Diabetes is a systemic metabolic disorder often accompanied by hypertension, hyperlipidemia, hyperuricemia, obesity, and heart failure, making it a major risk factor for cardiovascular disease [63]. One of the pathological cores of cardiovascular complications in diabetes is endothelial dysfunction caused by mitochondrial dysfunction and the resulting exacerbation of oxidative stress. Recent studies indicate that activation of TRPV1 plays a crucial role in improving diabetic vascular endothelial function, with its protective mechanism primarily involving two key signaling pathways: TRPV1/PKA/UCP2 and TRPV1/Ca^2+^/PGC-1α/OPA1 (Table 5).

On one hand, dietary capsaicin activates TRPV1 to improve endothelial mitochondrial dysfunction in diabetic and atherosclerotic mice by upregulating the PKA/UCP2 pathway [47,64]. UCP2 is an antioxidant protein localized to the inner mitochondrial membrane that reduces membrane potential through mild uncoupling, thereby decreasing ROS production [47]. It serves as a key downstream effector molecule of TRPV1. Studies confirm that capsaicin treatment inhibits high-glucose-induced upregulation of NAD(P)H oxidase and its p22phox subunit, while increasing p-eNOS levels in the aorta. This enhances NO bioavailability, reduces ROS accumulation, and ultimately improves endothelium-dependent vasodilation. This protective effect can be blocked by TRPV1 antagonists, PKA inhibitors, or UCP2 inhibitors. UCP2 knockout experiments further confirmed its indispensable role. Under high-glucose stimulation, capsaicin failed to improve endothelial dysfunction in aortic rings of UCP2 knockout mice, whereas it produced significant effects in wild-type mice [64]. Furthermore, this pathway enhances expression of the mitochondrial complex I subunit NDUFA9, restores complex I activity, improves mitochondrial oxidative phosphorylation and electron transport function, and mitigates excessive mitochondrial membrane potential polarization. However, these effects are absent in TRPV1 or UCP2 knockout models [47].

On the other hand, in a diabetic cardiac microvascular injury model, TRPV1 mitigates mitochondrial damage through the Ca^2+^/PGC-1α/OPA1 signaling axis [65]. A high-sugar, high-fat (HG-HF) environment suppresses TRPV1 expression and intracellular Ca^2+^ concentration in cardiac microvascular endothelial cells (CMECs). Following capsaicin activation of TRPV1, Ca^2+^ influx is promoted, thereby upregulating PGC-1α and its downstream mitochondrial fusion protein optic atrophy 1 (OPA1). This effect is absent in TRPV1 knockout cells and can be blocked by TRPV1 antagonists or Ca^2+^ chelators. Concurrently, HG-HF induces severe mitochondrial dysfunction in CMECs, manifested by decreased mitochondrial membrane potential, reduced ATP synthesis, and significantly elevated levels of ROS and nitrotyrosine (a marker of nitrosative stress). TRPV1 deficiency exacerbates these abnormalities. Supplementation with OPA1 partially reversed the aforementioned mitochondrial damage and oxidative/nitrosative stress. This suggests that under diabetic conditions, TRPV1 activates the PGC-1α/OPA1 axis via Ca^2+^ influx, with OPA1 acting as a key downstream molecule. By maintaining mitochondrial function and mitigating oxidative stress, OPA1 protects cardiac microvascular endothelial cells.

Notably, MAMs, as critical microdomains regulating mitochondrial calcium homeostasis and function, have garnered increasing attention for their role in diabetic complications. Studies have shown that in diabetic cardiomyopathy, a high-glucose environment inhibits the activity of AMPK, thereby relieving its inhibition on the downstream target gene Fundc1. This leads to upregulation of Fundc1 expression and promotes excessive MAM formation, ultimately resulting in mitochondrial calcium overload, dysfunction, and myocardial pathology, suggesting that the AMPK-Fundc1-MAM axis is an important pathogenic pathway [66]. Interestingly, previous research in diabetic nephropathy has revealed that activation of TRPV1 similarly upregulates AMPK through Ca^2+^ influx and inhibits Fundc1 transcription, thereby reducing MAM formation in podocytes and improving mitochondrial function and protein filtration barrier integrity [67]. Consistent with recent findings in cardiomyocytes—demonstrating that TRPV1 channels not only localize to MAMs but also directly participate in regulating Ca^2+^ exchange within this microdomain [44]—these lines of evidence collectively imply that the protective role of TRPV1 on mitochondrial function may extend beyond the classical scope of the vascular endothelium. The negative regulation of MAM formation by TRPV1 via the Ca^2+^/AMPK/Fundc1 signaling axis may represent a common organelle-level mechanism underlying its protective effects in multiple diabetic target organs, including the heart and kidney. This deserves further investigation. Therefore, targeting TRPV1 to modulate MAM-mediated mitochondrial homeostasis may offer a novel strategy for the prevention and treatment of a broader spectrum of diabetic cardiovascular complications, including diabetic cardiomyopathy.

In brief, among cardiovascular complications of diabetes, TRPV1 acts as a key molecule through two pathways—PKA/UCP2 and Ca^2+^/PGC-1α/OPA1—to exert endothelial protective effects by improving mitochondrial respiratory function and enhancing antioxidant defense mechanisms. Additionally, activation of TRPV1 negatively regulates the formation of MAMs via the Ca^2+^/AMPK/Fundc1 signaling axis, thereby preventing pathological mitochondrial calcium overload and dysfunction in diabetic nephropathy. This implies that TRPV1 may also play a crucial role in broader diabetic cardiovascular complications.

**Table 5:** The expression of TRPV1 in the cardiovascular system and its impact on diabetes.

References	Localization	TRPV1 Action	Experimental Model and Modeling Diseases	Impact	Pathway
Xiong et al. [47]	Aorta	Activation	TRPV1/ApoE/UCP2 knockout mice; Coronary artery dysfunction	Regulate mitochondrial function	TRPV1/PKA/UCP2
Sun et al. [64]	Aortic	Activation	WT, TRPV1 knockout, db/db and UCP2 knockout mice;mouse aortic rings; PIECs Diabetes	Regulate mitochondrial function	TRPV1/PKA/UCP2
Li et al. [65]	CMECs	Activation	WT, TRPV1 knockout mice, CMECs T2DM	Regulate mitochondrial function	TRPV1/Ca^2+^/PGC-1α/OPA1

TRPV1: transient receptor potential vanilloid 1; ApoE: Apolipoprotein E; PIECs: porcine iliac artery endothelial cells; WT: wild-type; PKA: protein kinase A; UCP2: Uncoupling protein 2; CMECs: cardiac microvascular endothelial cells; T2DM: Type 2 diabetes mellitus.

## 4. TRPV1 as a Therapeutic Target for Cardiovascular Diseases

### 4.1. Myocardial Infarction and Myocardial Ischemia–Reperfusion Injury

TRPV1 represents a significant potential target in the prevention and treatment of myocardial infarction and myocardial ischemia–reperfusion injury. Its effects are complex, with protective mechanisms exhibiting multi-pathway characteristics that are highly dependent on cellular location, activation intensity, and timing of intervention.

The TRPV1/CGRP axis occupies a pivotal position in mediating cardioprotective effects. In the context of myocardial ischemia–reperfusion injury, CGRP released upon TRPV1 activation confers protection through multiple mechanisms. As a potent vasodilator, CGRP promotes improved coronary microcirculation, an established aspect of its cardioprotective action. Additionally, experimental studies in myocardial infarction models have demonstrated that CGRP can directly attenuate the inflammatory response. For example, in TRPV1-deficient mice, administration of exogenous CGRP significantly reduced myocardial levels of pro-inflammatory cytokines (TNF-α, IL-6) and neutrophil infiltration, effects that were mechanistically associated with suppression of the NF-κB pathway [29]. Similarly, in a rat model of acute myocardial ischemia, enhancement of myocardial TRPV1/CGRP signaling via electroacupuncture pretreatment was shown to decrease NF-κB p65 protein expression and reduce infarct size [58]. These findings suggest that activation of TRPV1 on sensory nerve fibers promotes the release of neuropeptides such as CGRP, contributing to these protective effects.

Beyond neuromodulation, directly regulating the activity state of TRPV1 within cardiomyocytes themselves represents a crucial strategy. Studies confirm that low-concentration capsaicin activates TRPV1 to mitigate cellular injury and infarct size, yet this effect can be blocked by calcineurin inhibitors. This suggests that CAP exerts its action through a calcineurin-dependent mechanism within a narrow therapeutic window [14]. Calcineurin, as a calcium-dependent phosphatase, can reactivate TRPV1, and this process during the early phase of reperfusion may lead to calcium overload and exacerbated injury [14,68]. Under the pathophysiological conditions of ischemia–reperfusion, this reactivation may lead to excessive opening of TRPV1 channels and calcium overload, thereby exacerbating injury. Therefore, precisely regulating the activity level of TRPV1, particularly the channel state during the early reperfusion phase, is crucial for achieving cardiac protection. Based on the conserved interaction site between the C-terminal region of TRPV1 and the calmodulin A subunit, researchers synthesized the peptide V1-cal to specifically inhibit this interaction during early reperfusion. Experiments confirmed that V1-cal effectively reduced myocardial infarction size, demonstrating superior efficacy compared to either capsaicin or calmodulin inhibitors alone [14]. This indicates that indirect regulation of TRPV1 channels, rather than direct activation or antagonism, represents an effective strategy for avoiding calcium overload toxicity and achieving cardiac protection.

TRPV1 also serves as a key mediator in endogenous cardiac protection strategies, such as ischemic preconditioning and postconditioning. Myocardial ischemic preconditioning can be further categorized based on the timing and location of ischemia/reperfusion: preconditioning (PC) (stimulation delivered to the heart before sustained ischemia), ischemic postconditioning (IpostC) (stimulation delivered to the heart at the onset of reperfusion), remote preconditioning (RIPC) (stimulation delivered to distant tissues before sustained ischemia), and remote postconditioning (stimulation delivered to distant tissues at the onset of reperfusion) [24,69,70,71,72]. Research indicates that the cardioprotective effects induced by local or remote ischemic preconditioning and postconditioning are abolished following TRPV1 gene knockout or administration of its antagonists, CGRP, and substance P receptor blockers [73,74]. This indicates that TRPV1 activation promotes the release of CGRP and substance P from sensory neurons, representing a common downstream pathway for these endogenous protective strategies. Furthermore, the protective effects of remote pre-treatment are also associated with the inhibition of glycogen synthase kinase-3β activity and enhanced gap junction communication [75,76,77]. Notably, the protective effects of TRPV1 agonists exhibit clear concentration- and time-dependent properties. Low concentrations or short-term treatment activate protective pathways, whereas high concentrations or sustained activation may induce receptor desensitization or produce harmful effects, leading to the loss or even reversal of protective benefits [6,14]. Therefore, precise time-dependent control of its activity is crucial.

Additionally, TRPV1 can be activated by endogenous lipid mediators and participates in related pathways. Studies indicate that hypoxic pre-conditioning promotes the production of 12(S)-HETE, a metabolite of arachidonic acid 12-lipoxygenase (ALOX12), by upregulating ALOX12. As an endogenous ligand for TRPV1, 12(S)-HETE subsequently activates protein kinase C subtypes, thereby inducing cardioprotective effects. The ALOX12/TRPV1/PKC pathway can be blocked by TRPV1 or ALOX12 inhibitors [78].

Notably, TRPV1 regulation within the central nervous system also influences cardiac outcomes. Intrathecal administration of morphine inhibits TRPV1 activity in dorsal root ganglia (DRG) via activation of μ-opioid receptors, suppressing cAMP-PKA pathway-mediated signaling. This reduces TRPV1 protein expression and phosphorylation levels in DRG induced by ischemia–reperfusion injury, thereby decreasing myocardial infarction size and arrhythmia scores [7]. This suggests that inhibiting TRPV1 activity at sensory nerve afferents may exert a protective effect by modulating nociceptive signal transmission.

Taken together, TRPV1 plays a dual and complex role in myocardial ischemia–reperfusion injury, with its ultimate effects determined by the cellular location of action and the level of systemic integration. At sensory nerve endings, its activation primarily mediates the release of neuropeptides (such as CGRP and substance P), exerting remote protective effects. These effects are prominently characterized by vasodilation and, in the setting of cardiac ischemia, include significant anti-inflammatory actions. However, based on the hypothesis that TRPV1 may localize to mitochondria, its excessive activation within cardiomyocytes is proposed to directly contribute to calcium overload and mitochondrial dysfunction. The observation that precise modulation of TRPV1 activity (e.g., using V1-cal) can convert detrimental outcomes into benefits provides functional support for the aforementioned regulatory model involving TRPV1-calcineurin interaction. Therefore, therapeutic strategies targeting TRPV1 must precisely distinguish its cell-specific localization and functional context. By temporally controlling its activity levels or intervening in specific protein interactions, it can be transformed into a safe and effective cardioprotective target.

### 4.2. Hypertension

Increased sympathetic nervous system activity is a recognized hallmark of primary hypertension and a negative prognostic indicator [79]. Activation of TRPV1 leads to the release of neuropeptides from cardiac sensory nerve endings. These neuropeptides are transmitted to the central nervous system via spinal afferent nerves, triggering sympathetic nervous system excitation. Neuromodulation targeting thoracic spinal sensory nerves may represent a potential therapeutic approach for primary hypertension. Research has demonstrated that in spontaneously hypertensive rats (SHR), ablation of thoracic (T1–T4) TRPV1 neurons via RTX significantly reduces blood pressure in 16-week-old hypertensive rats and prevents further blood pressure elevation in 8-week-old SHR. However, the effects of RTX may be model-dependent and site-specific in hypertension. In the angiotensin II-induced hypertension model, RTX treatment failed to alter the blood pressure elevation trend. Furthermore, treating the lumbar (L2–L5) ganglia with RTX or applying RTX to the epicardium did not reduce blood pressure in adult SHR. Furthermore, findings from studies on renal vascular hypertension (2K1C model) indicate that TRPV1 channels play a critical role in renal sensory nerves. Their activation leads to increased renal afferent nerve activity, subsequently triggering sympathetic excitation and elevated blood pressure [80]. In male TRPV1 knockout rats, 2K1C surgery-induced hypertension was significantly attenuated, manifested by reductions in mean arterial pressure (MAP), systolic blood pressure, and diastolic blood pressure. Concurrently, renal afferent nerve activity and total renal sympathetic nerve activity decreased, while glomerular filtration rate improved. Notably, sex differences may play a crucial role in TRPV1’s effects on hypertension, as 2K1C-induced hypertension in female rats remained unchanged by TRPV1 deficiency. This suggests that inhibiting TRPV1 to reduce sympathetic nervous system excitation represents a promising therapeutic approach for treating both primary hypertension and renovascular hypertension.

### 4.3. Diabetes

Diabetic neuropathy, involving both central and peripheral nerves, ranks among the most common complications of diabetes. Damage to cardiac sympathetic afferent fibers plays a significant role in asymptomatic myocardial ischemia associated with diabetes. Activation of TRPV1 induces calcium influx, which in turn triggers phosphorylation of CaMKII and cAMP response element binding protein (CREB), ultimately upregulating the expression of cardioprotective neuropeptides such as CGRP. Under diabetic conditions, downregulation of TRPV1 and CGRP expression is closely associated with poor recovery after myocardial ischemia. Paeoniflorin (PF), a monoterpenoid glycoside extracted from *Paeonia*, exhibits multiple pharmacological activities including anti-inflammatory, antioxidant, immunomodulatory, and neuroprotective effects [81]. Research has revealed that PF pretreatment can significantly reduce myocardial infarction area following ischemia in diabetic mice, lower serum cardiac enzyme levels, and improve cardiac function. This occurs by upregulating TRPV1 expression, activating the TRPV1/CaMK/CREB signaling cascade, and promoting CGRP synthesis and release. Furthermore, TRPV1 gene knockout or treatment with the CaMKII inhibitor KN-93 attenuates the protective effects of PF, confirming that TRPV1 and its downstream signaling pathways play a crucial role in PF-mediated myocardial protection [82].

**Figure 2:**
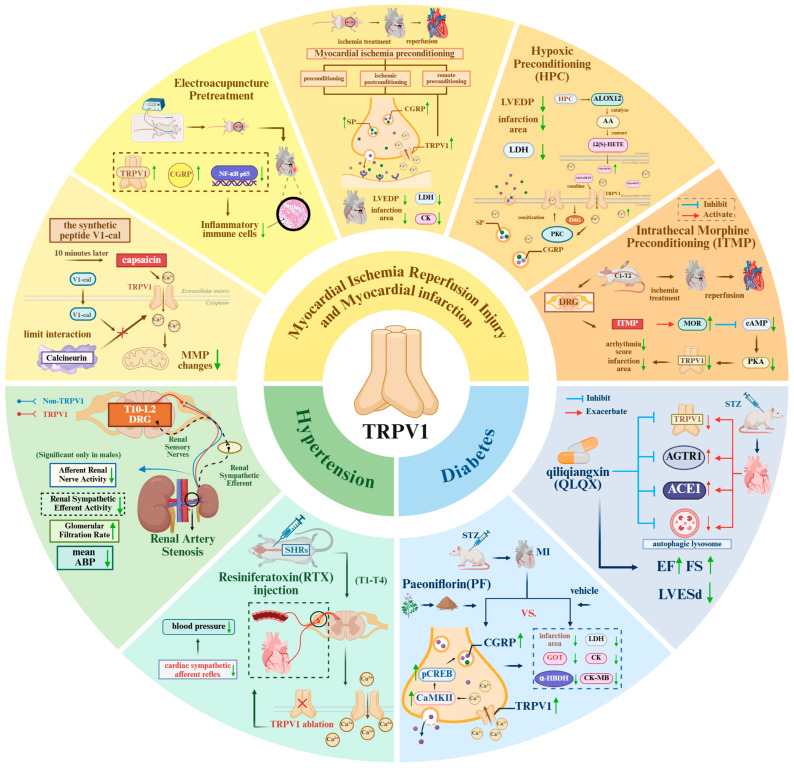
Mechanisms of TRPV1 as a Therapeutic Target in Myocardial Infarction, Myocardial Ischemia–Reperfusion Injury, Hypertension, and Diabetes. TRPV1, transient receptor potential vanilloid 1; MMP, mitochondrial membrane potential; CGRP, calcitonin gene-related peptide; NF-κB, nuclear factor kappa-B; SP, substance P; LVEDP, left ventricular end-diastolic pressure; LDH, lactate dehydrogenase; CK, creatine kinase; ALOX12, arachidonate 12-lipoxygenase; AA, arachidonate; 12(S)-HETE, 12(S)-hydroxyeico-satetraenoic acid; DAG, diacyl glycerol; PKC, protein kinase C; MOR, μ-opioid receptor; cAMP, cyclic adenosine monophosphate; PKA, protein kinase A; ABP, arterial blood pressure; STZ, streptozocin; CaMK, Ca^2+^/Calmodulin-dependent protein kinase; CREB, cAMP response element binding protein; GOT, glutamic-oxaloacetic transaminase; CK-MB, creatine kinase-MB; α-HBDH, α-hydroxybutyrate dehydrogenase; ROS, reactive oxygen species; AGTR1, angiotensin II receptor type 1; ACE1, angiotensin converting enzyme 1; EF, ejection fraction; FS, fractional shortening; LVESd, left ventricular end-systolic internal diameter.

Additionally, angiotensin-converting enzyme 1 (ACE1)/angiotensin II receptor type 1 (AGTR1) plays a pivotal role in diabetic cardiac dysfunction. ACE1 converts angiotensin I to angiotensin II, while AGTR1 serves as the primary receptor for angiotensin II. Its activation induces vasoconstriction, cell proliferation, fibrosis, and inflammatory responses. Studies in STZ-induced diabetic mouse models revealed that TRPV1 activation also improves diabetes-induced cardiac dysfunction by restoring autophagy in cardiomyocytes [83]. Compared with untreated diabetic mice, treatment with the traditional Chinese medicine Qili Qiangxin (QLQX) significantly improved diabetes-induced cardiac dysfunction by suppressing diabetes-induced upregulation of ACE1/AGTR1, restoring TRPV1 expression levels, and increasing the number of autophagosomes and autophagolysosomes. This manifested as increased ejection fraction (EF) and short-axis shortening fraction (FS), reduced LVESd, and alleviated myocardial fibrosis and hypertrophy. These findings suggest that modulating the AGTR1/TRPV1 pathway and its mediated autophagy may represent a novel strategy for preventing and treating diabetic cardiac dysfunction (Figure 2).

## 5. Discussion

This review provides a comprehensive synthesis of current advances regarding the role of TRPV1 in cardiovascular diseases, with a particular focus on the underlying mechanisms and signaling pathways. Previous studies have consistently demonstrated that TRPV1 channels serve as crucial modulators in myocardial infarction, ischemia–reperfusion (I/R) injury, adverse cardiac remodeling, heart failure, and related cardiovascular disorders. However, the outcomes of TRPV1 activation are highly dependent on the disease context, timing, and cellular localization, reflecting its complex functional versatility.

Our review highlights several important findings. First, activation of TRPV1 can attenuate cardiac dysfunction following myocardial infarction by inhibiting inflammatory responses and modulating fibroblast behavior. Importantly, in the scenario of I/R injury, TRPV1’s role appears to be double-edged: depending on the timing and cellular environment, its activation may produce either cardioprotective or deleterious effects. This duality is mediated by both neuropeptide signaling pathways, such as CGRP and SP, and direct regulation of cellular calcium and mitochondrial homeostasis. Prior work, supported by animal models and clinical observations, suggests that transient inhibition of the TRPV1-calcineurin interaction at the time of reperfusion (for instance, through peptide inhibitors like V1-cal) selectively curbs mitochondrial dysfunction and apoptosis while maintaining neural-mediated protective mechanisms. This finding may offer new opportunities for translational research and therapeutic development.

Further, beyond ischemic injury, TRPV1 has been shown to confer protection against pathological myocardial hypertrophy and contractile impairment through mechanisms including mitochondrial quality control, upregulation of SIRT3 and NDUFA9, as well as activation of the eNOS/NO pathway. In the context of cardiac remodeling, TRPV1 modulates oxidative stress, promotes mitochondrial biogenesis, and suppresses maladaptive fibroblast activation via TGF-β/Smad and PPAR-δ/UCP2-related signaling. Nevertheless, recent evidence indicates that excessive or dysregulated TRPV1 activation may exacerbate pathological remodeling or cellular injury, which underscores the importance of precise temporal and spatial context in TRPV1-targeted interventions.

Notably, TRPV1 also regulates sympathetic nervous system excitability, which forms the theoretical basis for its potential in managing heart failure, hypertension, diabetic cardiomyopathy, and their associated complications, primarily through anti-oxidative and anti-inflammatory mechanisms.

Taken together, these findings position TRPV1 as a novel integrative regulator in cardiovascular pathophysiology, bridging mitochondrial homeostasis, redox balance, neuroimmune interaction, and cardiac structural integrity. The most innovative insight from recent studies is the context-dependent dual role of TRPV1, highlighting the possibility of leveraging state- and localization-specific interventions—such as targeting the TRPV1-calcineurin axis—to optimize outcomes in I/R injury and cardiac remodeling. Moving forward, further research is needed to precisely characterize the spatiotemporal dynamics of TRPV1 activation, particularly regarding downstream neuropeptide-mediated signaling events, to advance the development of targeted and individualized therapeutic strategies for cardiovascular diseases.

## 6. Conclusions

Recent advances have highlighted the multifaceted roles of TRPV1 in cardiovascular disease, revealing it as an integrative regulator that links mitochondrial homeostasis, redox balance, neuroimmune interaction, and cardiac remodeling. The context-dependent effects of TRPV1 underscore its potential as both a protective and a pathogenic factor, determined by disease state, timing, and cellular localization. The dual actions of TRPV1, as evidenced in experimental and clinical studies, demonstrate the complexity of its signaling networks within the cardiovascular system.

Overall, targeting TRPV1 and its associated signaling pathways presents a promising avenue for precision therapy in cardiovascular diseases. Future research should focus on further delineating the spatiotemporal mechanisms of TRPV1 activation and interactions, as well as the downstream neuropeptide signaling events. This will facilitate the development of more targeted, individualized strategies for prevention and intervention, ultimately improving clinical outcomes for patients with cardiovascular disease.

## Data Availability

No new data were created or analyzed in this study.

## Abbreviations

The following abbreviations are used in this manuscript:

AB	Abdominal aortic banding
ACE1	Angiotensin-converting enzyme 1
Akt	Protein kinase B
ANP	Atrial natriuretic peptide
APD	Action potential duration
Bax	Bcl-2-associated X protein
Bcl-2	B-cell lymphoma 2
BH4	Tetrahydrobiopterin
BNP	Brain natriuretic peptide
CaMKII	Calmodulin-dependent protein kinase II
CGRP	Calcitonin gene-related peptide
CHF	Chronic heart failure
CI	Mitochondrial complex I
CMECs	Cardiac microvascular endothelial cells
CSAR	Cardiac sympathetic afferent reflex
CSNA	Cardiac sympathetic nerve activity
CTGF	Connective tissue growth factor
CVD	Cardiovascular disease
ECM	Extracellular matrix
eNOS	Endothelial nitric oxide synthase
ERK1/2	Extracellular signal-regulated kinase 1/2
ET-1	Endothelin-1
FS	Fractional shortening
GSK3β	Glycogen synthase kinase-3β
HF	Heart failure
H/R	Hypoxia/reoxygenation
HW/BW	Heart weight to body weight ratio
IL-1β, IL-6	Interleukin-1β, Interleukin-6
iNOS	Inducible nitric oxide synthase
IRI	Ischemia–reperfusion injury
LVEDD	Left ventricular end-diastolic diameter
LVEDP	Left ventricular end-diastolic pressure
LVEF	Left ventricular ejection fraction
LVESV	Left ventricular end-systolic volume
LVSP	Left ventricular systolic pressure
MAP	Mean arterial pressure
MCP-1	Monocyte chemoattractant protein-1
MI	Myocardial infarction
MIP-2	Macrophage inflammatory protein-2
MMP	Matrix metalloproteinase
mPTP	Mitochondrial permeability transition pore
NF-κB	Nuclear factor kappa-B
NK1	Neurokinin-1 receptor
NO	Nitric oxide
OPA1	Optic atrophy 1
PCI	Percutaneous coronary intervention
PGC-1α	Peroxisome proliferator-activated receptor-γ coactivator-1α
PI3K	Phosphatidylinositol 3 kinase
PIP2/PIP3	Phosphatidylinositol 4,5-bisphosphate/Phosphatidylinositol 3,4,5-trisphosphate
PKA/PKC	Protein kinase A/Protein kinase C
PPAR-δ	Peroxisome proliferator-activated receptor δ
ROS	Reactive oxygen species
RTX	Resiniferatoxin
RyR2	Ryanodine receptor 2
SHR	Spontaneously hypertensive rat
SIRT3	Sirtuin 3
SP	Substance P
TAC	Transverse aortic constriction
TGF-β1	Transforming growth factor-β1
TNF-α	Tumor necrosis factor-α
TOM20	Translocase of the outer mitochondrial membrane 20
TRPV1	Transient receptor potential vanilloid subtype 1
UCP2	Uncoupling protein 2
